# Treatment Response of Morphea Patients Referred to a Tertiary Dermatology Hospital: A Three‐Year Cohort Study

**DOI:** 10.1002/hsr2.71482

**Published:** 2025-11-05

**Authors:** Nika Kianfar, Mahan Babaahmadi, Shayan Dasdar, Seyed Mohammad Vahabi, Kimiya Ghiasvand, Zeinab Aryanian, Mahshid Sadat Ansari, Ifa Etesami

**Affiliations:** ^1^ Department of Dermatology, Razi Hospital, School of Medicine Tehran University of Medical Sciences Tehran Iran; ^2^ Autoimmune Bullous Diseases Research Center, Razi Hospital Tehran University of Medical Sciences Tehran Iran

## 1 Introduction

Morphea, also known as localized scleroderma, is an autoimmune disorder that affects the skin and underlying connective tissues. It typically begins with forming inflammatory plaques, which progress to fibrosis, causing thickening and stiffness of the skin and deeper tissues [1]. While mild cases are often managed with topical treatments, more severe forms require immunosuppressive drugs with anti‐inflammatory properties. Phototherapy also serves as an effective treatment option [2].

This study examines clinical features and treatment outcomes in morphea patients followed for at least 6 months, addressing gaps in research on disease course and treatment effectiveness. The findings offer valuable insights to enhance clinical management and improve outcomes for both dermatologists and patients.

## 2 Methods

### 2.1 Study Design

This prospective cohort study involved morphea patients treated at a tertiary dermatology hospital between 2021 and 2024, approved by the Tehran University of Medical Sciences ethics committee (IR. TUMS. MEDICINE. REC.1401.297). Patients were selected from our center's morphea registry, and all provided informed consent before participating. The diagnosis was made clinically, and confirmed with histopathologic findings in the majority of our cases.

### 2.2 Clinical Measures

Demographic and clinical characteristics were assessed at each visit, including physical examination findings, treatment type, and response. Disease severity was assessed using the Localized Scleroderma Cutaneous Assessment Tool (LoSCAT), a validated clinical instrument that measures disease activity through the Localized Scleroderma Activity Index (LoSAI) and tissue damage through the Localized Scleroderma Damage Index (LoSDI) [3].

### 2.3 Treatment Approaches

Patients were primarily managed with methotrexate. Mycophenolate mofetil was initiated in cases of inadequate response or intolerance to methotrexate. Prednisolone was commonly used as bridge therapy. Most patients also received adjunctive narrowband ultraviolet B (NB‐UVB) phototherapy. The treatments were tapered once disease control was achieved (Supporting Table 1).

### 2.4 Statistical Analysis

Only patients with follow‐up visits beyond 6 months were included in the statistical analysis. Disease severity was assessed at the baseline and follow‐up sessions. Patients who achieved a LoSAI of 0 were considered complete responders. Statistical analysis was performed using SPSS software (version 24, IBM, Chicago, USA), with significance defined as *p* < 0.05.

## 3 Results

### 3.1 Baseline Characteristics

This study evaluated 57 morphea patients (mean age 41.8 years) and predominantly female (91.2%). The average time from disease onset to diagnosis was 22.5 months. Generalized morphea was the most common subtype (52.6%), while patch and plaque lesions were the most frequently observed lesion pattern (64.9%). The limbs and trunk were the most common sites of involvement for 40 (70.2%) and 36 (63.2%) of patients, respectively. At baseline, the mean LoSAI score was 6.1 ± 6.4, the mean LoSDI was 18.0 ± 16.8, and the composite LoSCAT score was 24.1 ± 21.7, reflecting mild to moderate disease severity. Over half of the cohort (57.9%) had underlying conditions. Most patients (57.9%) received systemic treatment, while 21.1% were treated with a combination of systemic therapy and NBUVB phototherapy (Table 1).

**Table 1:** Baseline characteristics of 57 patients with morphea.

Variable		
Age, year, mean ± SD		41.8 ± 18.3
Gender, *n* (%)	Female	52 (91.2%)
	Male	5 (8.8%)
Underlying condition, *n* (%)	Positive^a^	33 (57.9%)
	Negative	24 (42.1%)
Presence of symptoms, *n* (%)	Positive^b^	19 (55.9%)
	Negative	15 (44.1%)
Time to diagnosis, month, mean ± SD		22.5 ± 46.9
Clinical variant, *n* (%)	Generalized	30 (52.6%)
	Plaque type	16 (28.1%)
	Linear	11 (19.3%)
Lesion type, *n* (%)	Patch and plaque	37 (64.9%)
	Atrophic	26 (45.6%)
	Sclerotic	17 (29.8%)
	Post‐inflammatory hyperpigmentation	12 (21.1%)
	hypopigmentation	8 (14.0%)
	Lilac ring	4 (7.0%)
	Linear	12 (21.1%)
	Alopecia	2 (3.5%)
Location, *n* (%)	Head and neck	16 (28.1%)
	Limbs	40 (70.2%)
	Trunk	36 (63.2%)
	Genital	2 (3.5%)
LoSAI, mean ± SD		6.1 ± 6.4
LoSDI, mean ± SD		18.0 ± 16.8
LoSCAT, mean ± SD		24.1 ± 21.7
Type of treatment, *n* (%)	Only topical	9 (15.8%)
	Systemic^c^	33 (57.9%)
	NBUVB	3 (5.3%)
	Systemic^c^ + NBUVB	12 (21.1%)
Prednisolone initial dose, mg/kg/day, median (min – max)		20 (5‐30)
MTX current dose, mg/week, mean ± SD		11.1 ± 3.5
MTX cumulative dose, mg, mean ± SD		519.2 ± 1067.2

Abbreviations: LoSAI, Localized Scleroderma Activity Index; LoSDI, Localized Scleroderma Damage Index; LoSCAT, Localized Scleroderma Cutaneous Assessment Tool; MTX, methotrexate; NBUVB, narrowband UVB phototherapy.

^a^ The underlying conditions of the patients were as follows: psoriasis, osteoporosis, rheumatoid arthritis, hypertension, hyperlipidemia, migraine, vitiligo, hyperthyroidism, hypothyroidism, diabetes mellitus, osteoporosis, anemia.

^b^ Itching (*n* = 14), Pain (*n* = 4), Tingling (*n* = 1).

^c^ Methotrexate (n = 44), Prednisolone (*n* = 15), Mycophenolate mofetil (*n* = 1).

### 3.2 Treatment Response

**Figure 1:**
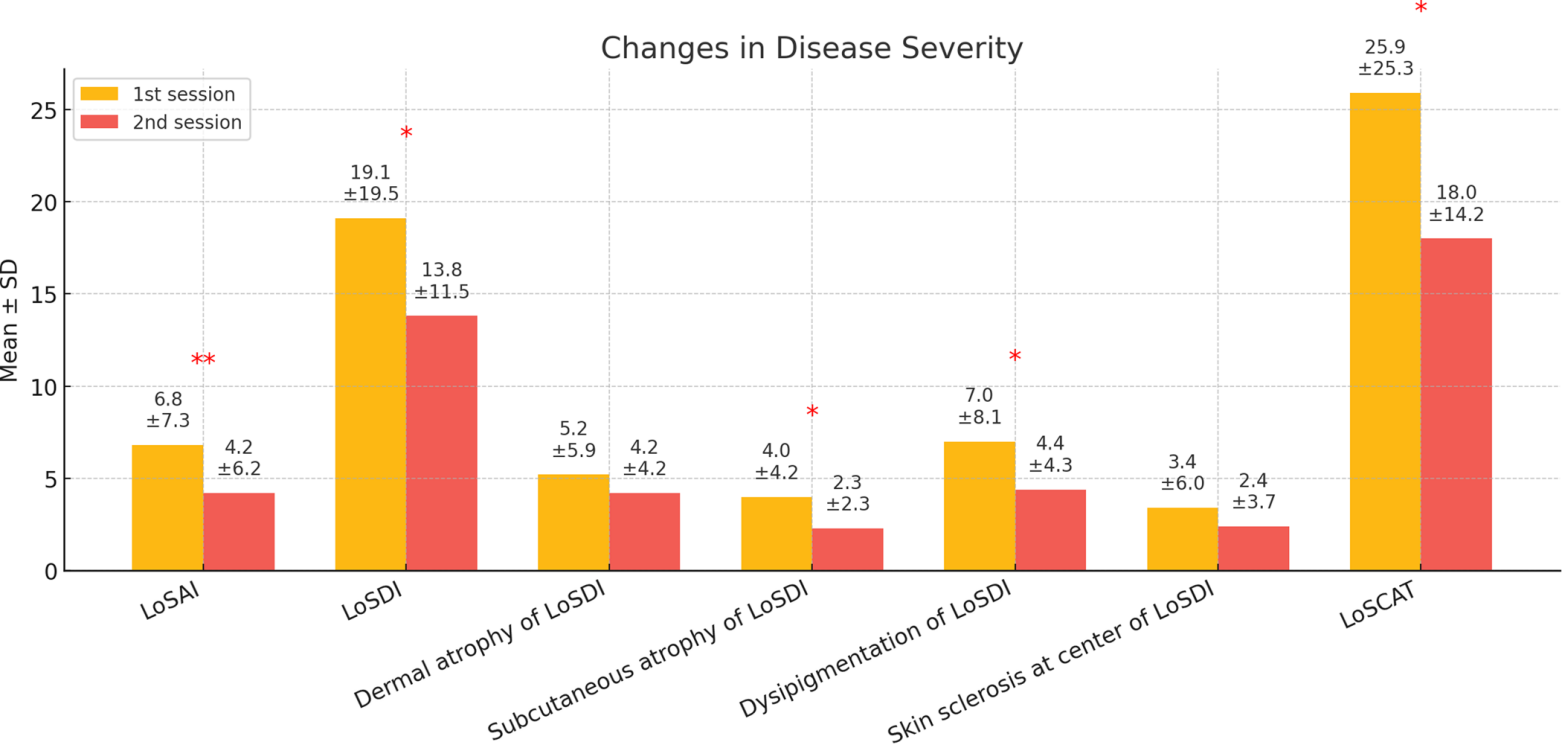
The changes in disease severity from baseline until 6 months after the start of treatment in 31 patients with more than 6 months of follow‐up. Dermal atrophy, Subcutaneous atrophy, Dyspigmentation, and Skin sclerosis are components of LoSDI. Significance level was measured based on Wilcoxon Signed Ranks Test, a non‐parametric test for paired data; *p < 0.05, **p < 0.01, ***p < 0.001. Abbreviations: LoSAI, Localized Skin Severity Index; LoSDI, Localized Scleroderma Damage Index; LoSCAT, Localized Scleroderma Cutaneous Assessment Tool.

Among the 31 (54.4%) patients with at least 6 months of follow‐up, improvement in LoSAI was observed in 24 (77.4%) patients. Of them, 10 (32.2%) demonstrated a complete response. The Wilcoxon Signed Ranks Test, a non‐parametric test for paired data, indicated significant decreases in LoSAI (*p* = 0.008), LoSDI (*p* = 0.039), and LoSCAT (*p* = 0.021) from baseline to follow‐up. Notably, while dermal atrophy and skin thickness changes were not statistically significant, subcutaneous atrophy (*p* = 0.033) and dyspigmentation (*p* = 0.038) showed significant improvement (Figure 1).

Regarding factors affecting the severity scale alterations, younger age was significantly associated with LoSAI reduction (‐0.386 per year, *p* = 0.032) and was marginally associated with LoSCAT reduction (−0.351 per year, *p* = 0.053) scores. Patients with head and neck involvement were less likely to experience a decrease in LoSAI (*p* = 0.012). Conversely, patients with trunk and limb involvement showed greater reductions in LoSAI (*p* = 0.008, *p* = 0.032) and LoSDI (*p* = 0.022, *p* = 0.002), respectively. To evaluate treatment outcomes, patients were classified into three groups based on their treatment: topical treatment only in 3 patients (9.7%), methotrexate alone in 18 patients (58.1%), and a combination of methotrexate and phototherapy in 10 patients (32.3%). Patients receiving combination therapy with methotrexate and phototherapy showed the greatest reduction in LoSAI scores (−6.6 ± 5.2), whereas those treated with topical therapy alone had the largest decrease in LoSDI (−10.3 ± 23.2). Changes in LoSCAT scores were variable across groups, with combination therapy again showing the largest mean decrease (−14.2 ± 18.0). However, none of these differences reached statistical significance (all *p* > 0.05) (Supporting Table 2).

## 4 Discussion

In this study, we enrolled 57 patients with morphea, 31 of whom had at least 6 months of follow‐up. The standard treatment administered to patients resulted in a marked improvement in the disease course. The treatment effectively controlled disease activity in 77% of patients, consistent with prior studies showing methotrexate efficacy at 80% [4], and phototherapy at 88% [5]. Although the combination of methotrexate and phototherapy resulted in better outcomes, the improvement was not statistically significant. Complete clinical response was observed in 32% of patients.

This study is one of the first to demonstrate a significant improvement in subcutaneous atrophy after 6 months of treatment, a novel and promising finding, given that subcutaneous atrophy is typically resistant to short‐term therapy. In our cohort, subcutaneous atrophy and dyspigmentation showed significant reductions, whereas dermal atrophy and dermal sclerosis remained largely unchanged over the follow‐up period. The lack of significant improvement in dermal atrophy is expected given the limited duration of observation. In contrast, the notable improvement in subcutaneous atrophy was unexpected. It may be partly attributed to the predominance of generalized morphea in our cohort, where patients often present with lesions at varying stages of activity and resolution, potentially allowing earlier visible changes.

In comparison, the O'Brien study, a prospective cohort of 102 patients, reported a complete response rate of 59% at 1 year [6]. While most patients in that study experienced improvement in dermal sclerosis after long‐term follow‐up (≥ 5 years), dermal and subcutaneous atrophy often remained stable or worsened. The differences in findings between the two studies may be attributed to subtype variation (generalized in our study vs. linear in O'Brien) and follow‐up duration (6 months vs. ≥ 1 year).

Our analysis identified significant relationships between patient or disease‐related characteristics and treatment outcomes in morphea. Younger patients showed more favorable responses, and lesions on the limbs and trunk improved more than those on the head and neck. The poorer response observed in the head and neck region may be attributed to the thinner skin and limited subcutaneous tissue in these areas, which may allow fibrosis to progress more rapidly and deeply. Moreover, most of the morphea lesions in the head and neck region are of the linear subtype, which generally exhibits a lesser degree of response compared to the plaque subtype. Additionally, cosmetic considerations can limit the aggressiveness and tolerability of a potent topical treatment in this region. Higher baseline disease severity was also associated with a diminished clinical response, consistent with the findings of O'Brien et al. [6].

This pilot study aimed to explore early clinical responses to treatment in morphea. The relatively small sample size and short 6‐month follow‐up provided insight into short‐term effects but limit the generalizability and strength of the findings. Conducting the study at a single center may also impact external validity, as variations in treatment protocols and patient populations can exist across different institutions. Additionally, a substantial proportion of patients were lost to follow‐up, potentially introducing bias. These limitations underscore the need for larger, multicenter studies with longer follow‐up durations and strategies to validate these preliminary findings.

## 5 Conclusion

This study demonstrated that standard treatment can effectively reduce disease activity in morphea within 6 months, with notable improvement in subcutaneous atrophy. Younger age, lower baseline disease severity, and limb/trunk involvement were associated with better outcomes.

## Author Contributions

**Nika Kianfar:** Conceptualization; Methodology; Software; Data curation; Investigation; Writing – review & editing; Writing – original draft; Project administration. **Mahan Babaahmadi:** Data curation; Conceptualization. **Shayan Dasdar:** Methodology; Software; Formal analysis; Investigation; Writing – review & editing; Writing – original draft. **Seyed Mohammad Vahabi:** Methodology; Visualization; Investigation; Data curation. **Kimiya Ghiasvand:** Conceptualization; Methodology; Project administration. **Zeinab Aryanian:** Conceptualization; Methodology; Software; Project administration; Supervision. **Mahshid Sadat Ansari:** Supervision; Data curation; Conceptualization; Methodology. **Ifa Etesami:** Conceptualization; Methodology; Software; Data curation; Supervision; Writing – review & editing; Writing – original draft.

## Conflicts of Interest

The authors declare no conflicts of interest.

## Transparency Statement

The lead author Ifa Etesami affirms that this manuscript is an honest, accurate, and transparent account of the study being reported; that no important aspects of the study have been omitted; and that any discrepancies from the study as planned (and, if relevant, registered) have been explained.

## Supporting information


**Supporting Table 1:** Treatment approaches in patients with morphea.

**Supporting Table 2:** Probable factors that affected reduction in LoSAI, LoSDI, and LoSCAT in 31 patients with more than six months of follow‐up.

## Data Availability

The data that support the findings of this study are available from the corresponding author upon reasonable request.
