# Supplementary material for: Treatment Response of Morphea Patients Referred to a Tertiary Dermatology Hospital: A Three‐Year Cohort Study
Source: Health Sci Rep. 2025 Nov 5;8(11):e71482. doi: 10.1002/hsr2.71482 (PMC12589186; doi:10.1002/hsr2.71482)
Supplement: Supplementary file 1 — Supporting Table 1: Treatment approaches in patients with morphea. [file HSR2-8-e71482-s001.docx]

| Treatment | Dosage and Administration | Indication for Use |
| --- | --- | --- |
| Methotrexate | 15 mg/m²/week (children), 15–25 mg/m²/week (adults), orally; switched to subcutaneous if gastrointestinal intolerance occurred | First-line systemic therapy |
| Mycophenolate mofetil | 10 mg/day, orally | Second-line option for methotrexate intolerance or insufficient response |
| Prednisolone | 0.5–1 mg/kg/day | Short-term bridge therapy |
| Narrowband UVB Phototherapy | 3 sessions per week with a 10% dose increase per session | Adjunctive therapy |

**Supplementary Table 1** Treatment approaches in patients with morphea
