# Supplementary material for: Treatment Response of Morphea Patients Referred to a Tertiary Dermatology Hospital: A Three‐Year Cohort Study
Source: Health Sci Rep. 2025 Nov 5;8(11):e71482. doi: 10.1002/hsr2.71482 (PMC12589186; doi:10.1002/hsr2.71482)
Supplement: Supplementary file 2 — Supporting Table 2: Probable factors that affected reduction in LoSAI, LoSDI, and LoSCAT in 31 patients with more than six months of follow‐up. [file HSR2-8-e71482-s002.docx]

**Supplementary Table 1** Treatment approaches in patients with morphea

**Supplementary Table 2** Probable factors that affected reduction in LoSAI, LoSDI, and LoSCAT in 31 patients with more than six months of follow-up

| Variable | | Reduction in LoSAI | P of reduction | Reduction in LoSDI | P of reduction | Reduction in LoSCAT | P of reduction |
| --- | --- | --- | --- | --- | --- | --- | --- |
| Age^a^ | Beta coefficient | -0.386 | **0.032** | -0.240 | 0.193 | -0.351 | 0.053 |
| Adult-child^b^ | Adult (n=26) | -4.1 ± 6.6 | 0.055 | -6.1 ± 14.9 | 0.305 | -10.19 ± 18.5 | 0.115 |
|  | Child (n=5) | 4.8 ± 11.7 |  | -1.0 ± 4.4 |  | 3.8 ± 13.7 |  |
| Gender^b^ | Female (n=28) | -1.5 ± 6.7 | 0.181 | -3.5 ± 12.5 | 0.065 | -4.9 ± 15.0 | 0.077 |
|  | Male (n=3) | -14.0 ± 12.8 |  | -22.0 ± 16.5 |  | -36.0 ± 27.1 |  |
| Time to diagnosis^a^ | Beta coefficient | -0.114 | 0.541 | -0.071 | 0.705 | -0.104 | 0.579 |
| Clinical variant^c^ | Plaque-type (n=7) | 0.9 ± 10.8 | 0.363 | 2.7 ± 8.3 | 0.085 | 3.6 ± 12.5 | 0.084 |
|  | Linear (n=5) | -0.4 ± 3.2 |  | -3.8 ± 5.7 |  | -4.2 ± 8.8 |  |
|  | Generalized (n=19) | -4.6 ± 7.6 |  | -8.6 ± 15.8 |  | -13.2 ± 20.2 |  |
| Site of involvement | | | | | | | |
| Head and neck^b^ | Yes (n=8) | 2.1 ± 10.4 | **0.012** | -1.9 ± 7.3 | 0.339 | 0.3 ± 13.9 | 0.172 |
|  | No (n=23) | -4.4 ± 6.6 |  | -6.4 ± 15.4 |  | -10.8 ± 19.1 |  |
| Limbs^b^ | Yes (n=24) | -4.5 ± 6.7 | **0.008** | -6.9 ± 15.1 | 0.115 | -11.4 ± 18.7 | **0.026** |
|  | No (n=7) | 3.6 ± 9.7 |  | 0.43 ± 5.1 |  | 4.0 ± 11.3 |  |
| Trunk^b^ | Yes (n=20) | -4.8 ± 7.3 | **0.032** | -9.4 ± 14.8 | 0.005 | -14.3 ± 18.9 | **0.002** |
|  | No (n=11) | 1.3 ± 8.3 |  | 2.4 ± 7.4 |  | 3.6 ± 10.3 |  |
| Genital^b^ | Yes (n=1) | -12.0 ± 0 | 0.194 | -5.0 ± 0 | 1.000 | -17.0 ± 0 | 0.452 |
|  | No (n=30) | -2.4 ± 8.1 |  | -5.3 ± 14.0 |  | -7.6 ± 18.6 |  |
| LoSCAT | Beta coefficient | -.577 | **0.001** | -0.773 | **<0.001** | -0.836 | **<0.001** |
| Type of treatment^c^ | Only topical (n=3) | -1.7 ± 2.3 | 0.919 | -10.3 ± 23.2 | 0.513 | -12.0 ± 25.1 | 0.546 |
|  | Methotrexate (n=18) | -0.7 ± 9.3 |  | -3.1 ± 11.5 |  | -3.8 ± 17.5 |  |
|  | Methotrexate and phototherapy (n=10) | -6.6 ± 5.2 |  | -7.6 ± 15.5 |  | -14.2 ± 18.0 |  |
| MTX cumulative dose^a^ | Beta coefficient | 0.136 | 0.567 | -0.119 | 0.616 | 0.004 | 0.985 |

^a^ Based on linear regression

^b^ Based on Mann–Whitney U test

^c^ Based on Kruskal-wallis test

^d^ Formulation for Delta calculation: ((2^nd^ session score– 1^st^ session score)/1^st^ session score)(%)

Abbreviations: LoSAI, Localized Skin Severity Index; LoSDI, Localized Scleroderma Damage Index; LoSCAT, Localized Scleroderma Cutaneous Assessment Tool.
